# A novel approach to inhibit HIV-1 infection and enhance lysis of HIV by a targeted activator of complement

**DOI:** 10.1186/1743-422X-6-123

**Published:** 2009-08-12

**Authors:** Yuanyong Xu, Chuanfu Zhang, Leili Jia, Cuirong Wen, Huihui Liu, Yong Wang, Yansong Sun, Liuyu Huang, Yusen Zhou, Hongbin Song

**Affiliations:** 1 Institute of Disease Control and Prevention, Academy of Military Medical Science, Beijing 100071, PR China; 2 302 Hospital of People's Liberation Army, Beijing 100039, PR China; 3 Chinese Center for Disease Control and Prevention, Department of Epidemiology, Beijing 100050, PR China; 4 State Key Laboratory of Pathogen and Biosecurity, Beijing Institute of Microbiology and Epidemiology, Beijing 100071, PR China

## Abstract

**Background:**

The complement system is one of the most potent weapons of innate immunity. It is not only a mechanism for direct protection against invading pathogens but it also interacts with the adaptive immunity to optimize the pathogen-specific humoral and cellular defense cascades in the body. Complement-mediated lysis of HIV is inefficient but the presence of HIV particles results in complement activation by the generation of many C3-fragments, such as C3dg and C3d. It has been demonstrated that activation of complement can enhance HIV infection through the binding of special complement receptor type 2 expression on the surface of mature B cells and follicular dendritic cells.

**Presentation of the hypothesis:**

Previous studies have proven that the complement-mediated antibody-dependent enhancement of HIV infection is mediated by the association of complement receptor type 2 bound to the C3 fragment and deposited on the surface of HIV virions. Thus, we hypothesize that a new activator of complement, consisting of a target domain (C3-binding region of complement receptor type 2) linked to a complement-activating human IgG1 Fc domain (CR2-Fc), can target and amplify complement deposition on HIV virions and enhance the efficiency of HIV lysis.

**Testing the hypothesis:**

Our hypothesis was tested using cell-free HIV-1 virions cultivated *in vitro *and assessment of virus opsonization was performed by incubating appropriate dilutions of virus with medium containing normal human serum and purified CR2-Fc proteins. As a control group, viruses were incubated with normal human serum under the same conditions. Virus neutralization assays were used to estimate the degree of CR2-Fc-enhanced lysis of HIV compared to untreated virus.

**Implications of the hypothesis:**

The targeted complement activator, CR2-Fc, can be used as a novel approach to HIV therapy by abrogating the complement-enhanced HIV infection of cells.

## Background

The human immunodeficiency virus (HIV) causes severe immune deficiency in humans and currently affects up to 42 million people worldwide. To date, there are no effective vaccines against HIV infection due to a number of issues. Firstly, there have been several recent failures of potential vaccine candidates in clinical trials. In 2003, two phase 3 trials using gp120 protein for vaccination that were aimed to raise sterilizing, antibody-mediated immunity, failed to protect vaccinees from HIV infection [1,2]. Another vaccine trial using a different strategy (V520 of Merck) was stopped prematurely in September 2007 due to evidence that vaccinees may have been more susceptible to HIV infection than placebo control individuals [3]. Secondly, no effective therapeutic approach for "curing" HIV infected individuals is currently under clinical investigation. Current therapies for HIV infection using highly active antiretroviral therapy (HAART) are not able to eliminate virus completely and complications of these therapies include severe side effects and viral resistance that may establish latent reservoirs of HIV.

The complement system is a key component of innate immunity and provides a first line of defense against invading pathogens that can bridge the innate and adaptive arms of the immune system [4,5]. It is not only a mechanism for direct protection against invading pathogens but also interacts with the adaptive immune system to optimize the pathogen-specific humoral and cellular defense cascade in the body, especially for viral pathogens. HIV, however, has evolved several mechanisms to evade complement-mediated lysis (CML) and exploit the complement system to increase viral infectivity [6]. Thus, in light of recent failures for vaccine design, the present study proposes an innovative approach to find a novel targeted activator of complement for the elimination of HIV.

## Presentation of the hypothesis

### Interaction of HIV with the complement system

HIV infection leads to the immediate activation of the complement system, even in the absence of HIV-specific antibodies. However, after seroconversion, the presence of HIV-specific antibodies triggers further activation of the classical complement pathway [7]. Antibodies that may enhance HIV infection *in vitro *were described shortly after HIV had first been isolated. Robinson *et al. *[8] found that sera from HIV-infected individuals enhance *in vitro *HIV infection of the complement receptor type 2 (CR2; CD21)-bearing T lymphoblastoid cell line, MT2. The same authors demonstrated that this enhancement was dependent on antibodies and mediated by complement and coined the term complement-mediated antibody-dependent enhancement (C-ADE) [9]. The mechanism of C-ADE has been investigated by several studies during the past two decades. As summarized by Robinson *et al. *[8], binding of antibody to gp41 initiates the complement cascade and leads to the deposition of the C3dg complement component on the virion. Opsonized viruses subsequently bind to CR2 distributed on mature B cells and follicular dendritic cells (FDC). Ultimately, the engagement of CR2 and CD4 receptors by opsonized virions leads to an increased rate of HIV spread through the tissue culture with a ten-fold increase in viral reverse transcriptase released into the culture medium and an increase in HIV genomic RNA [10]. In addition, evidence from *in vitro *and *in vivo *studies indicated that C-ADE occurs early in infection during the acute, high viremia phase [11,12].

Since complement activation is an extremely potent mechanism of the innate immune system and is potentially dangerous for host cells, it is tightly regulated. This regulation is mediated by proteins such as cell surface-like membrane cofactor protein (MCP), decay accelerating factor (DAF) and protectin (CD59), and the soluble factor H(fH) that can down-regulate complement activation at several stages of cascade and protect host cells from complement-mediated damage. The complement system is strongly activated upon infection by HIV but CML of viruses is inefficient [7,13]. The susceptibility of HIV to CML has been shown to be dependent on the expression of the MCP and CD59 complement-regulatory proteins on infected cells [14]. Since HIV acquires the host cell membrane and its associated membrane proteins (including MCP, DAF and CD59) during budding, there is an intrinsic resistance of the virus to CML [15,16]. Studies have shown that blocking DAF and CD59 on HIV with specific antibodies results in an increased sensitivity of both primary isolates and laboratory-strains of HIV to CML [17,18].

HIV infection results in the activation of the complement system, even in the absence of HIV-specific Abs [19] and results in the deposition of C3 fragments on the viral surface both *in vitro *[20] and *in vivo *[21]. HIV bound extracellularly to FDC in the germinal centers of lymph nodes represent the largest viral reservoir in HIV-infected individuals [22,23]. The binding of this infectious pool of HIV in the germinal centers depends mainly on interactions of CR2 expressed on FDC (or B cells) with C3d fragments attached to the viral surface [21,24]. In addition, an association of complement-opsonized HIV with peripheral B cells through CR2-C3d interactions has been described in HIV-infected individuals [25]. These CR2-C3d interactions between B cells and HIV are critical for efficient B cell-mediated transmission of complement-opsonized HIV to T cells [26].

### Complement receptor type 2 on target and bystander cells

Complement activation by the presence of HIV particles results in the generation of many C3-fragments that are recognized by different complement receptors expressed on various cell types [5]. Among these, C3dg and C3d serve as ligands for CR2 with high affinity [19]. Binding of C3d-coated particles induces a temperature-dependent aggregation of CR2 in lipid rafts on cells. The cross-linking of CR2 and the B-cell receptors through complement-opsonized antigens decreases the threshold necessary for B-cell activation and contributes to a prolongation of B-cell antigen receptor signaling. Thus, CR2 plays an important role in B-cell activation and combines the innate and adaptive arms of the immune system. The CR2 on FDC can bind opsonized immune-complexes (ICs) and is important for B cell affinity maturation and the development of B-cell memory. A major mechanism of FDC trapping is binding of antibody and complement-opsonized HIV to CR2 [24,27]. Viruses retained in this way, even in the presence of neutralizing antibodies, have been shown to remain infectious *in vivo *for months and comprise a viral archive that can be transmitted to T cells and other target cells migrating through germinal centers. Similarly, infection of CD4+ T cells is facilitated by circulating B cells that carry HIV bound to CR2. This mode of trans-infection occurs with virus opsonized with complement alone or with complement plus antibody [20,28].

### The hypothesis

Recent findings have generated renewed interest in so-called "non-neutralizing" antibodies that are unable to directly inhibit free virus entry into target cells, but nonetheless, exhibit antiviral activity mediated by the Fc region of the antibody molecule. These antibody effector mechanisms include complement binding and viral lysis, phagocytosis of antibody-coated virions, and antibody-dependent cellular cytotoxicity [29,30]. The complement system constantly interacts with HIV during all stages of infection highlighting the importance of CR2 in C-ADE. Taken together, the hypothesis presented here investigates a new strategy using a fusion protein to target and amplify complement deposition on HIV virions regardless of modulating complement inhibitor expression. The fusion protein consists of target domain, the C3-binding region of CR2, linked to a complement-activating human IgG1 Fc domain (CR2-Fc). The novel complement activator, CR2-Fc, is expected to enhance complement deposition and result in the further production of CR2 ligands through the complement-activating Fc domain. Thus, CR2-Fc will down-regulate complement inhibitors (MCP, DAF and CD59) or block their function on HIV virions that may enhance CML. More importantly, this targeted complement activator is able to bind to sites of complement activation, so it is likely to improve their efficacy while reducing potentially serious side effects resulting from complement activation. Furthermore, the human IgG1 Fc domain can also play a role of fixing complement system, so the more complement activation that occurs will lead to more CR2-Fc targeting to HIV. Subsequently, the positive feedback loop generated by the complement cascade results in enhanced lysis of HIV and preventing infection of naïve cells.

## Testing the hypothesis

After preparation of human CR2-Fc fusion protein, biodistribution studies were performed to evaluate the biologic activity of CR2-Fc *in vitro*. HIV-1 was cultivated in H9 cells and cell-free virus obtained from supernatants. Infection experiments were performed in 24-well plates in triplicate and virus opsonization was performed by incubating appropriate dilutions of HIV in culture medium with normal human serum (NHS) and purified CR2-Fc proteins. A control group included viruses that were incubated with NHS only under the same conditions. Finally, neutralization tests were used to estimate the efficiency of CR2-Fc-enhanced lysis of HIV compared to controls.

## Implication of the hypothesis

A successful test of the hypothesis would demonstrate that CR2-Fc can bind to HIV virions and can result in an amplification of the complement activation cascade. As a consequence of this action, HIV would likely be eliminated by CML and further infection by HIV should be inhibited. Furthermore, CR2-Fc bound to HIV virions is likely to reduce potential damage of host cells and tissues resulting from excess complement activation. Thus, it is meaningful to investigate the potential role of CR2-Fc for the abrogation of HIV infection in humans, as this new finding would suggest a novel approach for HIV therapy.

## Competing interests

The authors declare that they have no competing interests.

## Authors' contributions

YYX, CFZ, LLJ and HBS prepared the paper. CRW, HHL, YW, YSS, LYH and YSZ participated in developing the hypothesis and collaborated in writing and reviewing of the article. All authors read and approved the final manuscript.
